# Native T1 mapping in patients with idiopathic dilated cardiomyopathy for the assessment of diffuse myocardial fibrosis: validation against histologic endomyocardial biopsy

**DOI:** 10.1186/1532-429X-17-S1-O84

**Published:** 2015-02-03

**Authors:** Yoshitaka Goto, Masaki Ishida, Shiro Nakamori, Motonori Nagata, Yasutaka Ichikawa, Kakuya Kitagawa, Kaoru Dohi, Masaaki Ito, Hajime Sakuma

**Affiliations:** 1 Radiology, Mie University Hospital, Tsu, Japan; 2 Cardiology, Mie University Hospital, Tsu, Japan

## Background

Late gadolinium enhancement (LGE) MRI provides a significant impact on prognosis in dilated cardiomyopathy (DCM) patients. However, LGE MRI is less suitable for quantifying the degree of fibrosis in diffusely diseased myocardium. T1 mapping technique allows for the quantitative assessment of extracellular volume fraction (ECV), which has been histologically validated against the collagen volume fraction (CVF). Native myocardial T1 also has a potential for the noninvasive detection of myocardial fibrosis. Recent study demonstrated that native myocardial T1 permits the discrimination between normal and diffusely diseased myocardium accurately in DCM patients. However, in-vivo histological validation of native myocardial T1 in DCM patients is still lacking. The aim of this study was to histologically validate native myocardial T1 for the assessment of diffuse myocardial fibrosis in DCM patients.

## Methods

Twenty DCM patients (18 men, 56.8±15.7 years old) underwent CMR including cine, LGE MRI and pre- and post-contrast T1 mapping using a modified Look-Locker inversion recovery sequence at 3T. A significant coronary artery disease was excluded by invasive coronary angiography in all patients. All patients underwent endomyocardial biopsies from right septal ventricle. The diagnosis of DCM was confirmed histologically in all patients. T1 values were quantified within the septal myocardium and LV blood pool with a heart rate correction. ECV was calculated from myocardial and blood T1 measured before and after the administration of gadolinium contrast medium and hematocrit measures. CVF was quantified histologically from biopsy specimens using picrosirius red staining.

## Results

**Figure 1:**
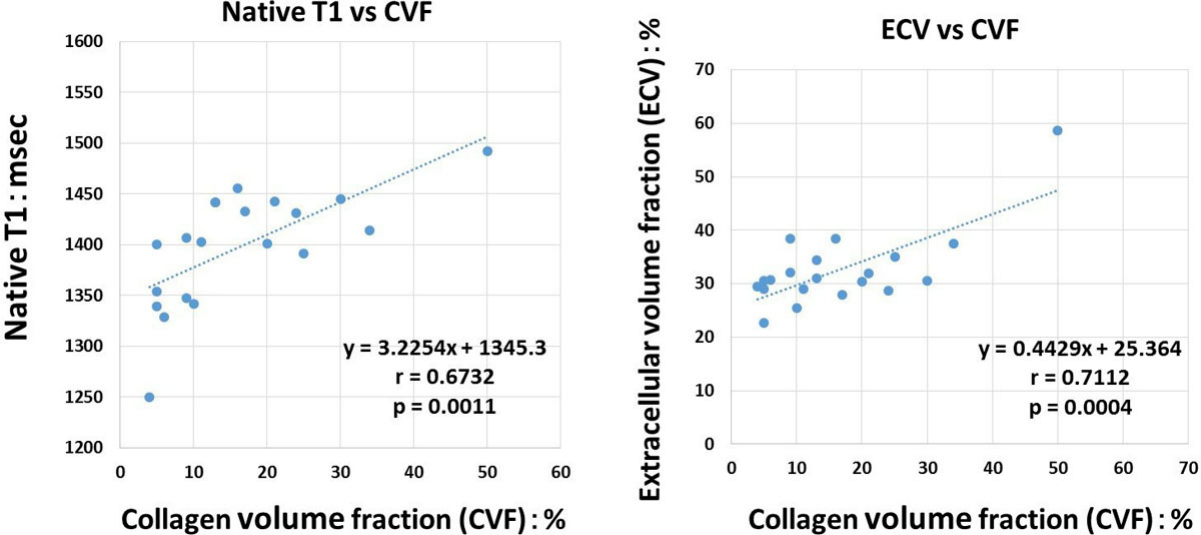


The CVF, native T1 and ECV was 16.4±11.5% (range, 4% to 50%), 1398.0±55.1ms (range, 1249.9 ms to 1491.8 ms) and 32.6±7.2% (range, 22.7% to 58.7%), respectively. LGE was observed in 5 of the 20 patients on LGE MRI. Both CVF and native T1 were significantly greater in the patients with LGE than those without LGE (CFV, 27.4 ±15.0 vs 12.7±8.1%, p=0.011; native T1, 1446.8±29.3 ms vs 1381.7±54.3 ms, p=0.021). Although the ECV tended to be grater in the patients with LGE than those without LGE, the difference was not significant (39.6±111.4% vs 30.3±3.6%, p=0.147). Both ECV and native T1 were strongly associated with CVF (y=0.443x+25.4, r=0.711, p<0.001 and y=3.225x+1345.3, r=0.673, p=0.001, respectively). Inter- and Intra- observer reproducibility for nativeT1 and ECV were 0.903, 0.977, 0.942 and 0.988, respectively. Figure 1.

## Conclusions

The current results demonstrated that both native T1 and ECV have a good correlation with histological collagen fraction in DCM patients. Native T1 mapping may serve as a noninvasive technique that allows for quantitative assessment of diffuse myocardial fibrosis in DCM patients without administrating gadolinium contrast medium.

## Funding

N/A.

